# Successful limb salvage in progressive proximal tibia osteosarcoma following denosumab chemotherapy: A case report

**DOI:** 10.1097/MD.0000000000029812

**Published:** 2022-07-29

**Authors:** Qian Chen, Junjie Wu, Kai Zheng, Ming Xu, Ziwei Hou, Xiuchun Yu

**Affiliations:** a First Clinical Medical College, Shandong University of Traditional Chinese Medicine, Jinan, Shandong, China; b Weifang Medical University, Weifang, Shandong, China; c Department of Orthopedics, The 960th Hospital of the People’s Liberation Army, Jinan, Shandong, China.

**Keywords:** chemotherapy, denosumab, osteosarcoma, RANK, RANKL

## Abstract

**Rationale:**

Osteosarcoma (OS) is a primary malignant bone tumor that originates in the mesenchymal tissue. It is the most common type of pleomorphic tumor occurring in children and adolescents. Currently, there is no established systematic treatment for OS that progresses during standard preoperative chemotherapy.

**Patient concerns and diagnoses:**

We describe a 14-year-old male patient with a 4-month history of pain in the upper right leg. Based on the results of percutaneous biopsy, a diagnosis of OS was made. After admission, the patient was treated with first-line chemotherapy agents. After a single course of treatment, the tumor progressed locally and no limb salvage was feasible.

**Interventions and outcomes:**

Intervention with denosumab combined with chemotherapy led to a significant reduction in tumor volume and ossification of soft tissue, which successfully resulted in limb salvage rather than amputation. The patient showed no evidence of recurrent or distant metastasis at 6-month follow-up.

**Lessons:**

Treatment with receptor activator of nuclear factor-ĸB ligand inhibitor denosumab combined with standard chemotherapy is effective for advanced OS progressing after chemotherapy. We recommend denosumab therapy for successful limb salvage in patients with high-grade OS associated with osteolytic bone destruction and refractory to preoperative neoadjuvant chemotherapy.

## 1. Introduction

Osteosarcoma (OS) is the most common type of primary malignant bone tumor in younger individuals. The pathophysiology of OS is characterized by inactivation of retinoblastoma and/or p53 tumor suppressor genes.^[1]^ Curative treatment for high-grade OS entails surgery of the primary tumor and chemotherapy in order to facilitate limb salvage and extend disease-free survival.^[2]^ Doxorubin, cisplatinum, high-dose methotrexate, and ifosfamide are considered the most active agents against OS. Several studies reported that the combination of chemotherapy and surgery increase the long-term survival rates above 70% and is widely accepted by physicians and patients.^[3]^ It is crucial to identify and validate new agents that might be administered alone or as adjuvants to conventional chemotherapy to improve not only patients’ chances of survival and cure but also their quality of life.^[4]^

In recent years, some primary bone tumors have been identified as new therapeutic targets for denosumab, particularly those expressing the receptor activator of nuclear factor-ĸB ligand (RANKL) and those involving osteoclast-mediated bone resorption. We report a 14-year-old male with proximal tibia OS who achieved successful limb salvage following treatment with denosumab combined with neoadjuvant chemotherapy. Also, we discuss the relevant clinicopathology, diagnosis, and treatment following the patient’s informed consent.

## 2. Case presentation

**Figure 1.**
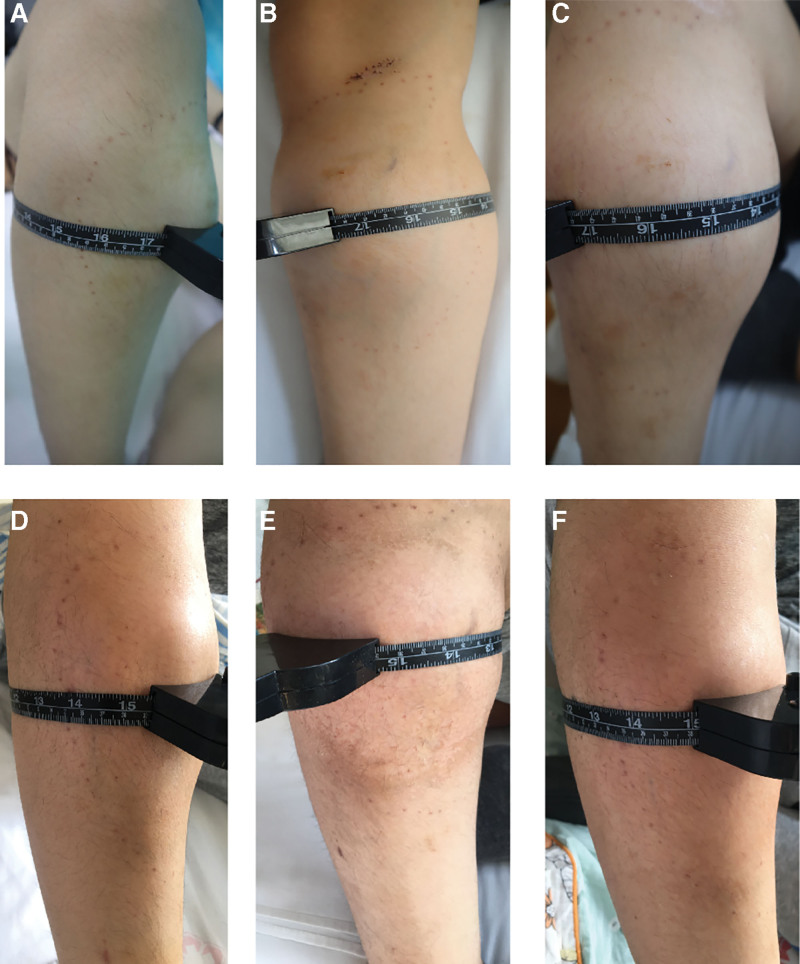
The maximum diameter of right leg tumor measured before chemotherapy was 44.2 cm (A), increasing to 44.5 cm after the first course of neoadjuvant chemotherapy (B). The diameter after the first dose of denosumab was 44.1 cm (C), 39.3 cm after the second dose of denosumab (D), and 39.0 cm after the third dose of denosumab (E). The circumference measured again before operation was 38.3 cm (F).

**Figure 2.**
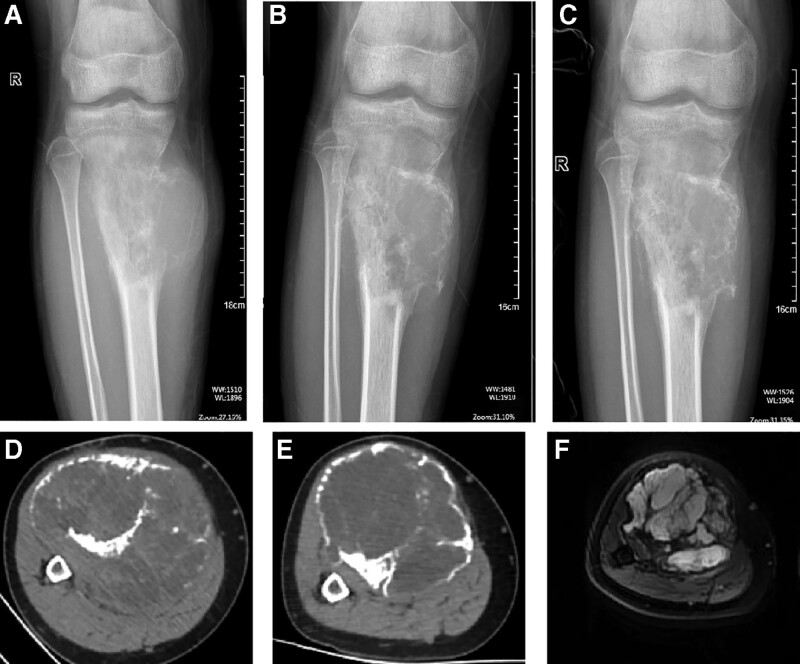
Plain radiograph: Before chemotherapy, the density of the proximal right tibia decreased, with a huge soft tissue mass surrounding it and an unclear boundary (A). The soft tissue volume was significantly reduced and the calcification was obvious after the addition of denosumab (B and C). Computed tomography images before (D) and 3 times after the administration of denosumab combined with chemotherapy (E).

A 14-year-old healthy male reported no obvious cause of pain involving his upper right leg in March 2021. At first, the pain was aggravated during activity and relieved at rest, accompanied by local swelling. After discussion with his family, imaging procedures and percutaneous biopsy were performed in April 2021. The pathology report showed telangiectatic OS. The patient refused surgical treatment and instead managed with traditional Chinese medicine and needles, without any efficacy. The tumor volume increased gradually. The patient was referred to our hospital for systematic treatment. Physical examination revealed a swollen proximal right leg. The eye of the puncture needle was visible; however, no change in skin color or superficial veins was seen. A palpable 10 × 8 × 3 cm firm mass was detected on his right leg, with elevation in local skin temperature, obvious tenderness, and no range of motion. Flexion and extension of the right knee joint were obviously limited by pain, and the maximum circumference of the tumor surface on the right was thicker than on the left side (Fig. 1A). Plain radiography of the right tibia and fibula showed swelling, irregular osteolytic bone destruction and soft tissue mass, uneven density, scattered irregular strips of tumor bone shadow, with associated bone cortical interruption and obvious periosteal reaction (Fig. 2A). Because of the pain and the large size of the tumor, the patient maintained a forced flexion position after admission and was not screened via magnetic resonance imaging. Computed tomography (CT) of the chest showed no evidence of pulmonary metastasis.

**Figure 3.**
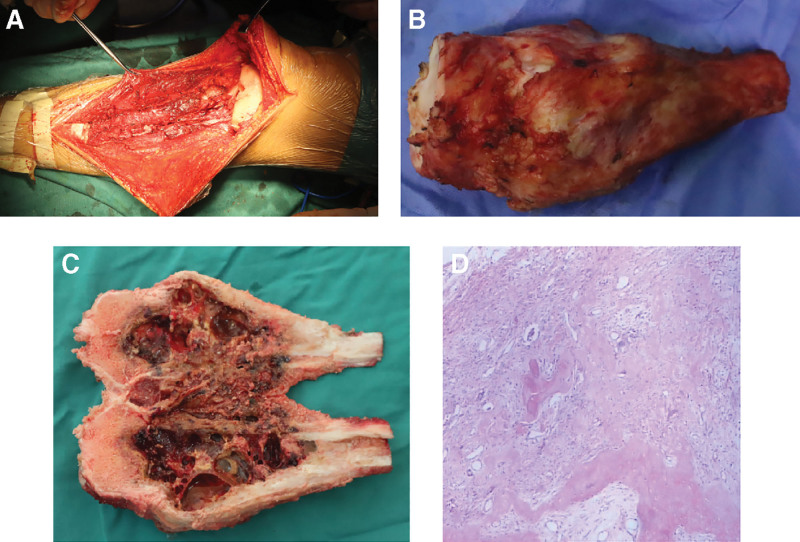
Intraoperatively, the tumor tissue was completely resected and the major neuro-vascular bundles and muscles were preserved, and no amputation was performed (A). Completely resected tumor tissue (B). Incised specimen tissue (C). The pathology results showed the presence of degenerative malignant tumor cells in all the tissues (D) (HE, ×100).

**Figure 4.**
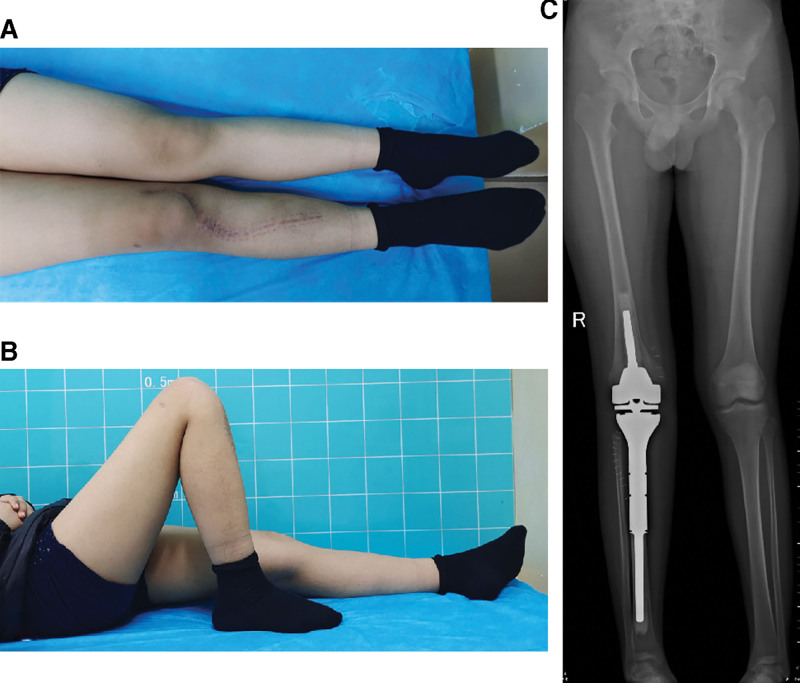
The incision healed well after operation (A). The function and activity of the right knee joint are basically normal (B). The postoperative plain radiograph showed good prothesis location without loosening; the small head of the right fibula was absent, and the length of both lower limbs was basically the same (C).

The patient was diagnosed with stage II B OS based on surgical staging system recommended for bone and soft tissue tumors. Overcoming chemotherapy taboos, the patient received a single neoadjuvant chemotherapy DIA regimen comprising cisplatin 120 mg/m^2^ for 1 day, doxorubicin liposome 60 mg/m^2^ for 1 day, and ifosfamide 2 g/m^2^ for 5 days. The maximum circumference of the tumor surface on the right proximal tibia was thicker than before chemotherapy (Fig. 1B). The tumor showed progression and no limb salvage was possible. Based on the imaging findings of osteolytic bone destruction, denosumab therapy was considered as it inhibits osteolysis and has been cleared by FDA for use in various conditions. Two weeks later, a decision was made to try DIA regimen combined with denosumab (120 mg, days 1, 8, and 15), in an effort to achieve limb salvage. Medications were routinely supplemented with vitamin D3 (600 µg daily). The patient had no side effects or complications. The maximum circumference of the right tumor measured again after each denosumab administration was smaller than that after the first course of neoadjuvant chemotherapy (Fig. 1C through F). Preoperative plain radiographs (Fig. 2B, C) and CT (Fig. 2E) showed that the volume of soft tissue mass was significantly reduced. Soft tissue and medullary calcification and ossification were found. Magnetic resonance imaging (Fig. 2F) revealed irregular polycystic expansive bone destruction in the metaphysis of the right tibia, without breaking through the epiphyseal plate. Excluding invasion of the surrounding soft tissue and capsule formation, a clear boundary and absence of adhesion to peripheral nerves and vessels were detected, which facilitated limb salvage surgery. In the junction area, the images showed circular and flaky isometry on T1 images and slightly longer on T2. On September 14, 2021, resection of the tumor and prosthesis reconstruction in the right proximal tibia was performed (Fig. 3A). The surgery was successful and the incision healed well (Fig. 4A). Postoperative pathology confirmed OS and a large necrotic tumor, along with degenerative malignant tumor cells in medullary cavity tissue, bone cortex, and extraosseous soft tissues. No tumor was found at the broken end (Fig. 3D). After 3 weeks, postoperative chemotherapy was performed sequentially (the dose and regime of chemotherapeutic drugs were the same as before operation) and functional exercise of the right lower limb was performed. Notably, denosumab was not added again during postoperative chemotherapy.

Six months after the operation, a total of 6 courses of adjuvant chemotherapy with DIA regimen were completed at an interval of 3 weeks. The patient’s Musculoskeletal Tumor Society score was 27 (Fig. 4B). The patient had no evidence of recurrent or distant metastasis based on CT of the chest. The postoperative plain radiography showed that the prothesis was in good position (Fig. 4C).

## 3. Discussion

According to the 2020 NCCN classical OS diagnosis and treatment guidelines, successful limb salvage surgery is based on safe surgical boundaries and good chemical reactions. Amputation is recommended (level 1) for patients undergoing preoperative neoadjuvant chemotherapy with unclear tumor boundaries and invasion of blood vessels and nerves. Studies have reported that the expression of RANKL in OS ranges from 8.8% to 75%.^[5–7]^ In the study of Bago-Horvath et al and Mori et al,^[7,8]^ a positive expression of RANK in OS was detected in 57% and 69% of patients, respectively. RANKL is a key factor that is required for osteoclast differentiation and activation.^[9]^ Some studies involving OS animal models^[10]^ have shown that anti-RANKL agents can effectively decrease tumor growth, improve survival and inhibit lung metastasis. Branstetter et al^[5]^ reported that anti-RANKL therapy may prevent osteolytic bone destruction in OS. A phase II study investigated denosumab alone in patients with relapsing/refractory OS was inadequate in this setting.^[9]^ In patients who are nonresponders to conventional chemotherapy, combining neoadjuvant chemotherapy regimens with targeted agents, such as RANKL-directed antibodies might improve survival.^[9]^ Denosumab is a human monoclonal antibody that inhibits bone resorption, increases new bone formation and delays tumor progression by binding to RANKL and preventing its interaction with RANK, thus mimicking the action of osteoprotegerin.

Punzo et al^[11]^ discouraged the use of denosumab in addition to conventional chemotherapy in OS, when used in combination with doxorubin, since it reduces its activity and probably inhibits the efficacy of the chemotherapy drug. However, the study did not classify the cases of standard OS based on imaging criteria, suggesting obvious limitations.

The imaging examination of the 14-year-old patient reported in our study showed that the lesion area of the proximal tibia was dominated by osteolytic bone destruction, which was mediated by the combination of RANKL and RANK. The binding of RANKL to RANK triggers osteoclast activation, which disrupts the balance of ecological environment in bone, and consequently prevents bone resorption and osteolysis. The results of preoperative plain radiograph reveal a time lag in osteogenesis after the application of denosumab. Given the limitations of a case report, a large number of clinical trials are needed to validate this phenomenon.

To our knowledge, no published reports are available to demonstrate successful limb salvage in OS with the addition of denosumab combined with first-line chemotherapy to achieve tumor progression during neoadjuvant chemotherapy. Therefore, for patients with high-grade OS associated with mainly osteolytic bone destruction based on imaging findings and the progress of preoperative neoadjuvant chemotherapy, denosumab can be used as a new agent in limb salvage surgery.

### Author contributions

Q.C. did the study, analyzed the data, and wrote the manuscript.

J.W. measured the tumor circumference, K.Z., M.X., Z.H., and X.Y. were involved in the design, data management, and analysis of the study. All authors read and approved the final manuscript.

Investigation: Q.C. and J.W.

Supervision: K.Z., M.X., Z.H., and X.Y.

Writing—original draft: Q.C.

Writing—review and editing: Q.C.

## References

[1] Savvidou OD Bolia IK Chloros GD. Denosumab: current use in the treatment of primary bone tumors. Orthopedics. 2017;40:204–10. 28732103 10.3928/01477447-20170627-04

[2] Piperno-Neumann S. Prise en charge des ostéosarcomes en 2009 [Diagnosis and treatment of primary osteosarcoma in 2009]. Bull Cancer. 2010;97:715–21. 20483711 10.1684/bdc.2010.1123

[3] Yuan G Chen J Wu D. Neoadjuvant chemotherapy combined with limb salvage surgery in patients with limb osteosarcoma of Enneking stage II: a retrospective study. Onco Targets Ther. 2017;10:2745–50 28603424 10.2147/OTT.S136621 PMC5457035

[4] Meazza C Bastoni S Scanagatta P. What is the best clinical approach to recurrent/refractory osteosarcoma? Expert Rev Anticancer Ther. 2020;20:415–28. 32379504 10.1080/14737140.2020.1760848

[5] Branstetter D Rohrbach K Huang LY. RANK and RANK ligand expression in primary human osteosarcoma. J Bone Oncol. 2015;4:59–68 27556008 10.1016/j.jbo.2015.06.002 PMC4986823

[6] Lee JA Jung JS Kim DH. RANKL expression is related to treatment outcome of patients with localized, high-grade osteosarcoma. Pediatr Blood Cancer. 2011;56:738–43. 21370405 10.1002/pbc.22720

[7] Bago-Horvath Z Schmid K Rössler F. Impact of RANK signalling on survival and chemotherapy response in osteosarcoma. Pathology. 2014;46:411–5. 24842377 10.1097/PAT.0000000000000116

[8] Mori K Le Goff B Berreur M. Human osteosarcoma cells express functional receptor activator of nuclear factor-kappa B. J Pathol. 2007;211:555–62. 17323424 10.1002/path.2140

[9] Beristain AG Narala SR Di Grappa MA. Homotypic RANK signaling differentially regulates proliferation, motility and cell survival in osteosarcoma and mammary epithelial cells. J Cell Sci. 2012;125(Pt 4):943–55. 22421365 10.1242/jcs.094029

[10] Chen Y Di Grappa MA Molyneux SD. RANKL blockade prevents and treats aggressive osteosarcomas [published correction appears in Sci Transl Med. 2016 Feb 3;8(324):324er2]. Sci Transl Med. 2015;7:317ra197. 10.1126/scitranslmed.aad0295 26659571

[11] Punzo F Tortora C Argenziano M. Can denosumab be used in combination with doxorubicin in osteosarcoma? Oncotarget. 2020;11:2763–73 32733647 10.18632/oncotarget.27669 PMC7367655

